# Supplementary material for: Machine Learning Models That Integrate Tumor Texture and Perfusion Characteristics Using Low-Dose Breast Computed Tomography Are Promising for Predicting Histological Biomarkers and Treatment Failure in Breast Cancer Patients
Source: Cancers (Basel). 2021 Nov 29;13(23):6013. doi: 10.3390/cancers13236013 (PMC8656976; doi:10.3390/cancers13236013)

Figure S1. Perfusion and texture analyses on low-dose breast CT in luminal and triple-negative cancers.

(Panel A) Perfusion and texture analyses on low-dose breast CT in a 58-year-old woman with invasive ductal cancer of the left breast (ER+, PR+, HER2-, Ki67-, low grade, and luminal A cancer).

A. Perfusion analysis of entire tumor

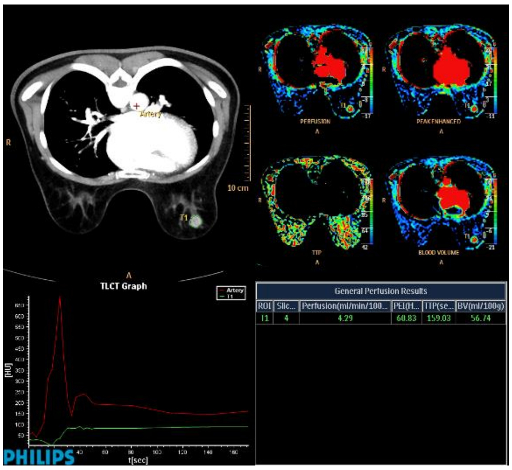

B. Perfusion analysis of hot spot

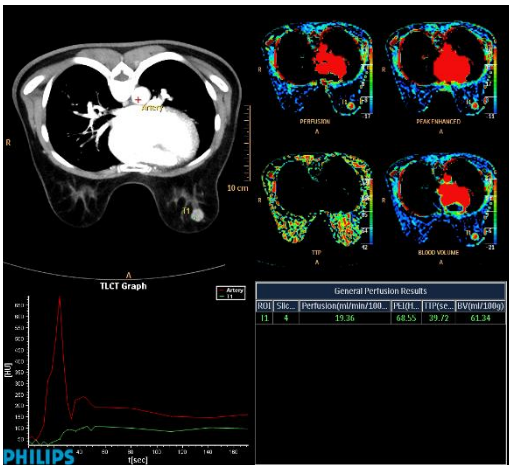

C. Texture analysis on precontrast image

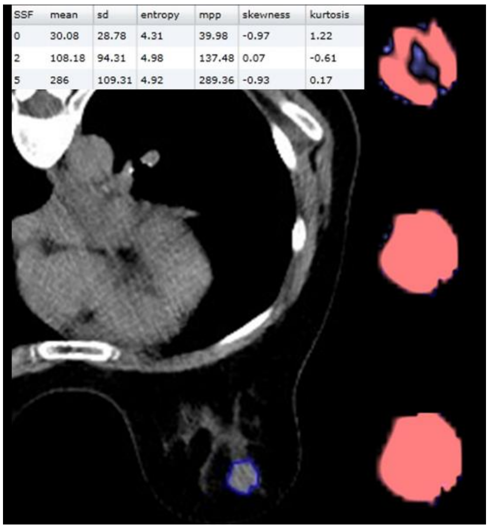

D. Histogram on precontrast image at SSF 0

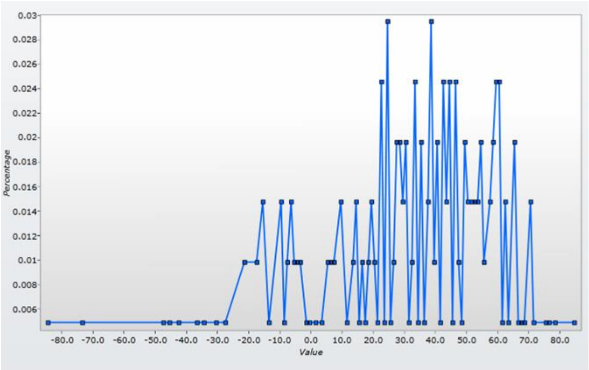

E. Texture analysis on contrast-enhanced image

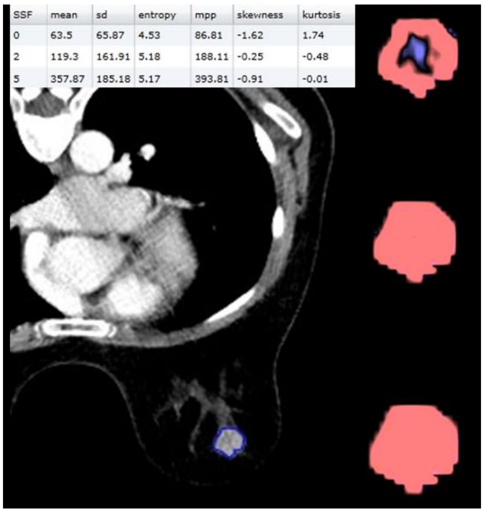

F. Histogram on contrast-enhanced image at SSF 0

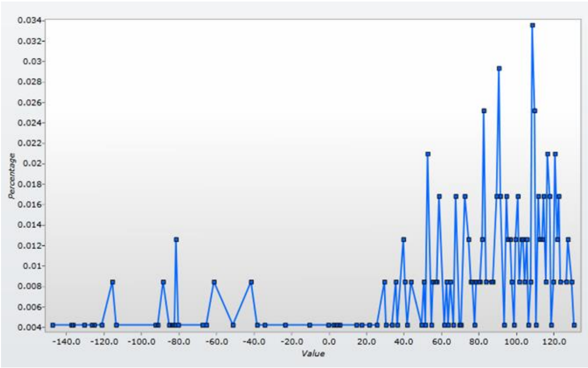

(Panel B) Perfusion and texture analyses on low-dose breast CT in a 52-year-old woman with invasive ductal cancer of the right breast (ER-, PR-, HER2-, Ki67+, high grade, and triple-negative cancer).

A. Perfusion analysis of entire tumor

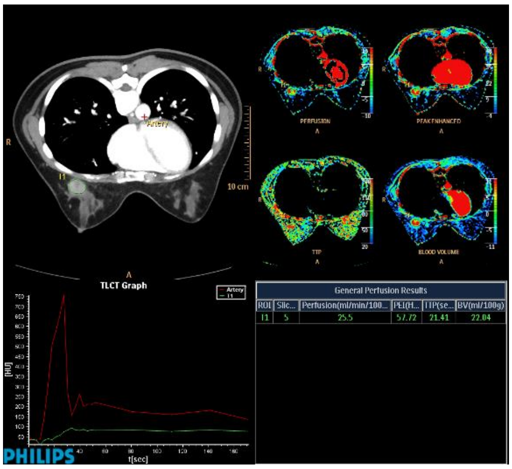

B. Perfusion analysis of hot spot

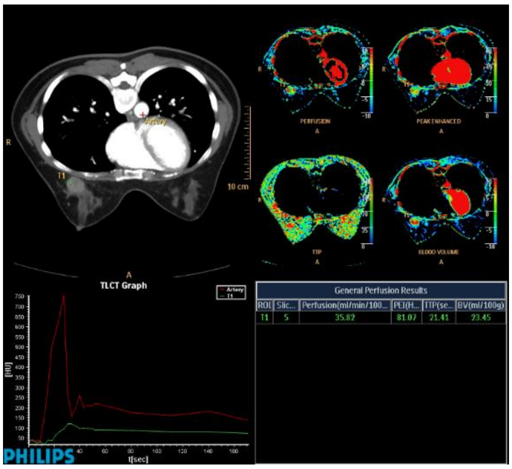

C. Texture analysis on precontrast image

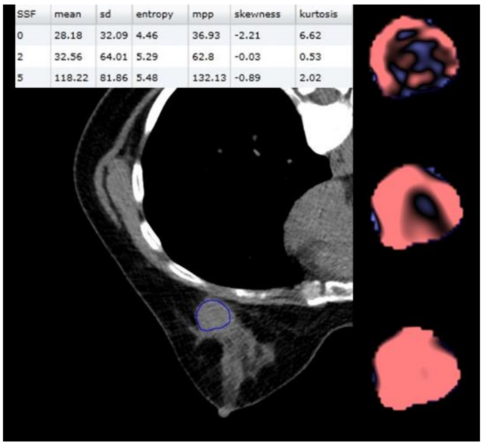

D. Histogram on precontrast image at SSF 0

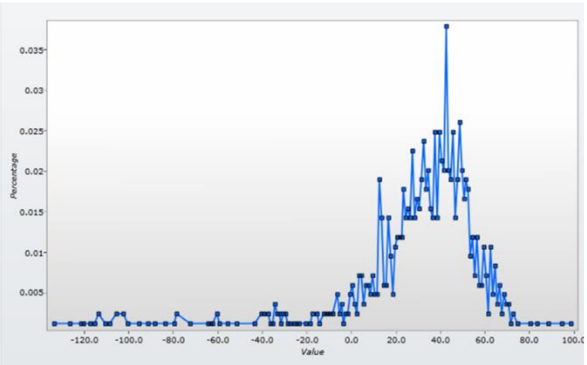

E. Texture analysis on contrast-enhanced image

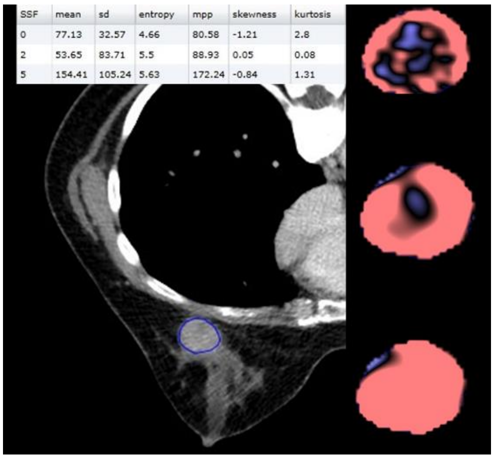

F. Histogram on contrast-enhanced image at SSF 0

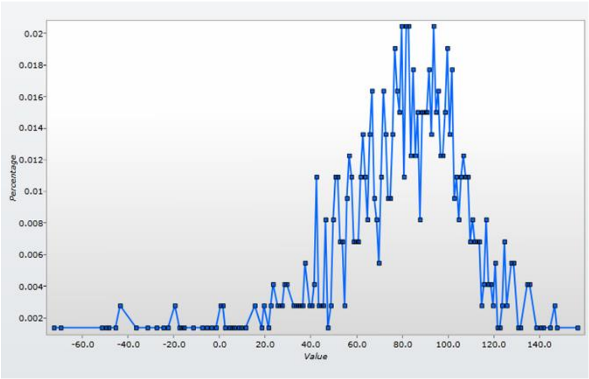

Supplement: Supplementary file 1 [file cancers-13-06013-s001.zip › Supplementary figure(Figure S1).pdf]
